# A two‐sample Mendelian randomization analysis of heart rate variability and cerebral small vessel disease

**DOI:** 10.1111/jch.14316

**Published:** 2021-07-01

**Authors:** Danyang Tian, Linjing Zhang, Zhenhuang Zhuang, Tao Huang, Dongsheng Fan

**Affiliations:** ^1^ Department of Neurology Peking University Third Hospital Beijing China; ^2^ Department of Epidemiology & Biostatistics School of Public Health Peking University Beijing China

**Keywords:** cerebral small vessel disease, heart rate variability, Mendelian randomization, white matter hyperintensity

## Abstract

Cerebral small vessel disease (cSVD) is correlated with a high risk of stroke and cognitive impairment. Previous studies between heart rate variability (HRV) and cSVD revealed paradoxical results. The authors aimed to investigate the relationship between HRV and cSVD using Mendelian randomization analysis. Genetic instruments for HRV were obtained from previous genome‐wide association studies. They applied inverse variance‐weighted analysis, weighted median analysis, simple median analysis, and Mendelian randomization–Egger regression to evaluate the associations of HRV with white matter hyperintensity (WMH) and small vessel stroke (SVS) in the UK Biobank neuroimaging dataset and the MEGASTROKE genome‐wide association study dataset. Two genetically predicted traits of HRV (the root mean square of the successive differences of inter beat intervals [RMSSD] and the peak‐valley respiratory sinus arrhythmia or high frequency power [pvRSA/HF]) were suggestively associated with WMH (β 0.26, 95% confidence interval [CI] 0.04–0.49, *p* = .02; β 0.14, 95% CI 0.02–0.27, *p* = .03, respectively). Genetically predicted traits of HRV were not significantly associated with SVS. This study provides genetic support for a suggestive causal effect of HRV (RMSSD, pvRSA/HF) on WMH but not SVS.

## 1 INTRODUCTION

Cerebral small vessel disease (cSVD) is correlated with a high risk of stroke and cognitive impairment.^[^ ^1^ ^]^ Despite abundant literature on the subject, there is still a lack of knowledge on the pathogenetic mechanisms underlying the development of cSVD, and part of the leaving knowledges are inconsistent. Recent studies have focused on several nontraditional risk factors of cSVD etiology and progression. Autonomic dysfunction is one of the nontraditional risk factors.^[^ ^2^ ^]^ One study showed that autonomic function, which is tested through abnormal blood pressure change in the Valsalva test, is significantly associated with neurological progression in patients with acute lacunar infarction.^[^ ^3^ ^]^ In a case‐control study, cSVD patients displayed significantly higher autonomic dysfunction indicators, such as 24‐h mean systolic BP (SBP), 24‐h mean diastolic BP (DBP), daytime mean SBP, nocturnal mean SBP, and nocturnal mean DBP. Heart rate variability (HRV) is an indicator that reflect autonomic dysfunction.^[^ ^4^ ^]^ Observational studies showed that HRV, which reflects the balance between sympathetic and parasympathetic tone, is associated with white matter hyperintensity (WMH), provided support for a relationship between autonomic dysfunction and cSVD. However, the results of previous studies were paradoxical.^[^ ^5^, ^6^ ^]^ A previous study found that HRV during the night was significantly higher in people with the progression of cSVD (including WMH and SVS [small vessel stroke]) than that of those without progression of cSVD in community‐dwelling elderly people.^[^ ^5^ ^]^ However, another study found no relation between HRV and WMH, and some found that low nighttime HRV was associated with a greater cSVD burden.^[^ ^6^ ^]^

Mendelian randomization (MR) is increasingly being used to examine the causal relationship between exposure and outcomes.^[^ ^7^ ^]^ Genetic alleles are randomly assorted during conception, and thus, confounding bias and reverse causation, which exist in observational studies, are avoided. If we did not observe pleiotropy in the approaches we use, a significant association in an MR study between exposure and outcome implies causality.^[^ ^8^, ^9^ ^]^ We aimed to investigate the genetic relationship between HRV and cSVD using MR analysis in the present study.

## 2 MATERIALS AND METHODS

### 2.1 Study design

**FIGURE 1:**
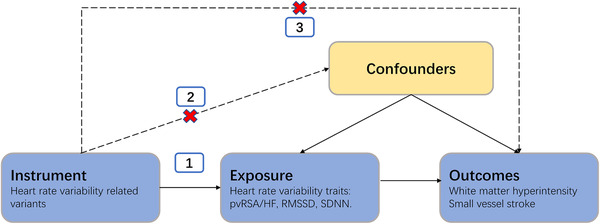
Schematic representation of the Mendelian randomization analysis. Broken lines represent potential pleiotropic or direct causal effects between variables that would violate Mendelian randomization assumptions. Abbreviations: pvRSA/HF: the peak‐valley respiratory sinus arrhythmia or high frequency power; RMSSD, the root mean square of the successive differences of interbeat intervals; SDNN, the standard deviation of the normal‐to‐normal interbeat intervals

This study follows the three assumptions of MR studies.^[^ ^10^ ^]^ (Figure 1) First, the genetic variant selected as an instrumental variable is associated with HRV; second, the genetic variant is not associated with any unmeasured confounders; third, the genetic variant is associated with cSVD only through HRV, not through other pathways. We applied a two‐sample MR analysis, which allowed selection of genetic variants as instruments for a risk factor (HRV) in one sample and explored associations of these variants with outcomes (cSVD) in another sample.^[^ ^10^, ^11^ ^]^

### 2.2 Data sources

Summarized data for HRV are available from published a genome‐wide association (GWAS) study.^[^ ^12^ ^]^ Three indices for HRV are used: the standard deviation of the normal‐to‐normal inter beat intervals (SDNN), the root mean square of the successive differences of inter beat intervals (RMSSD), and the peak‐valley respiratory sinus arrhythmia or high frequency power (pvRSA/HF). We examined the associations of the selected instruments with WMH volume in a GWAS analysis that we undertook in the UK Biobank neuroimaging dataset^[^ ^13^ ^]^ and with SVS in the GWAS summary statistics of the MEGASTROKE Consortium.^[^ ^14^ ^]^ The WMH data were derived from UK Biobank. Procedures for brain imaging acquisition and initial quality check have been described previously and are available on the UK Biobank website (Brain Imaging Documentation V1.3, http://www.ukbiobank.ac.uk).^[^ ^13^ ^]^ In the MEGASTROKE Consortium, all cases were ischemic stroke and had brain imaging to confirm this and to exclude a hemorrhage. The Trial of Org 10172 in Acute Stroke Treatment (TOAST) classification system was used for stroke subtyping identifying 2947 large artery strokes, 2757 small vessel strokes, and 3860 cardioembolic strokes. In addition, there were 8912 undetermined strokes (cases in which the stroke mechanism had not been identified) and multiple‐pathologies strokes. Genotyping was performed with a variety of Illumina or Affymetrix platforms. All cohorts underwent genotype imputation with the 1000 Genomes (1KG) phase I reference panel before meta‐analysis.^[^ ^14^ ^]^ The detailed information was listed on Table 1.

**TABLE 1:** Description of the genome‐wide association study of exposures and outcomes

	Consortium or study	Sample size	Age (y)	Men (%)	Ancestry	Year
Exposure						
pvRSA/HF	VgHRV	53174	54±5	54.9	European	2017
RMSSD	VgHRV	53174	54±5	54.9	European	2017
SDNN	VgHRV	53174	54±5	54.9	European	2017
Outcome						
SVS	MEGASTROKE	2191 cases, 27297 controls	65±14	54.5	Multiancestry	2018
WMH	UK Biobank	8429	62±7	48%	European	2018

Abbreviations: MEGASTROKE, the MEGASTROKE consortium; pvRSA/HF, the peak‐valley respiratory sinus arrhythmia or high frequency power; RMSSD, the root mean square of the successive differences of interbeat intervals; SDNN, the standard deviation of the normal‐to‐normal interbeat intervals; VgHRV, the genetic variance in heart rate variability consortium.

Since all analyses were based on publicly available summary statistics, no patients were involved in the design of the study, and no ethical approval from an institutional review board was required.

### 2.3 Genetic variants

We selected single nucleotide polymorphisms (SNPs) associated with HRV at a genome‐wide significance threshold (*p* < 5 × 10^–8^) in the published GWAS study^[^ ^12^ ^]^ (supplement Table S1). We conducted linkage disequilibrium (defined as *r* ^2 ^< 0.01) with other genetic variants to ensure independent genetic variants. When we encountered linkage disequilibrium, the variant with the lowest *p* value for association with the risk factor was selected. In instances where SNPs were not available in a data set because of poor imputation quality, we replaced them with proxy SNPs if available (*r* ^2 ^> 0.9).

### 2.4 Statistical analysis

We applied two‐sample MR analyses based on association estimates derived from the abovementioned sources. The inverse variance weighted (IVW) was conducted to estimate the causal relationship between HRV and cSVD. Sensitivity analyses such as the weighted median, simple median, and MR‐Egger regression were further conducted.^[^ ^15^, ^16^ ^]^ We used MR‐Egger and MR‐PRESSO analysis to evaluate the pleiotropy effects.^[^ ^10^ ^]^ Using the MR‐Egger method, the SNP's effect upon each exposure is plotted against its effect upon outcomes, and an intercept distinct from the origin provides evidence for pleiotropic effects (*p* for MR‐Egger <.05). A leave‐one‐out analysis was used to investigate the influence of outlying and/or pleiotropic genetic variants.^[^ ^17^ ^]^ The strength of the genetic instruments was tested with the F‐statistic through the website: http://cnsgenomics.com/shiny/mRnd/.^[^ ^18^ ^]^ All SNPs for the same exposure showed a strong association (*F*‐statistic > 10).

We conducted the statistical analysis using R version 3.3.3 (R foundation). A Bonferroni‐corrected significance threshold of *p* = .0083 (where *p* = .05/6 [three exposures and two outcomes]) was prespecified to adjust for multiple testing. Associations with *p* values between .05 and .0083 were considered suggestive evidence of a possible association.

## 3 RESULTS

### 3.1 Characteristics for selected SNPs

The characteristics of selected SNPs for each proxies of HRV are presented in supplement Table SI. We tested whether any of the selected SNPs were influenced by linkage disequilibrium (LD). We chose the variant with the lowest *p* value for association with each exposure, if genetic variants are in LD. Our genetic analysis showed that 1.32% of pvRSA/HF, 1.64% of RMSSD, and 1.41% of SDNN were explained by its SNPs. MR power calculation (http://cnsgenomics.com/shiny/mRnd) showed that we have 96%, 88%, 83% power to test significant (*p* < .05) causal effect of pvRSA/HF, RMSSD, and SDNN on WMH, and we have 48%, 96%, 78% power to test significant (*p* < .05) causal effect of pvRSA/HF, RMSSD, and SDNN on SVS, respectively.

### 3.2 The association between HRV and WMH

**FIGURE 2:**
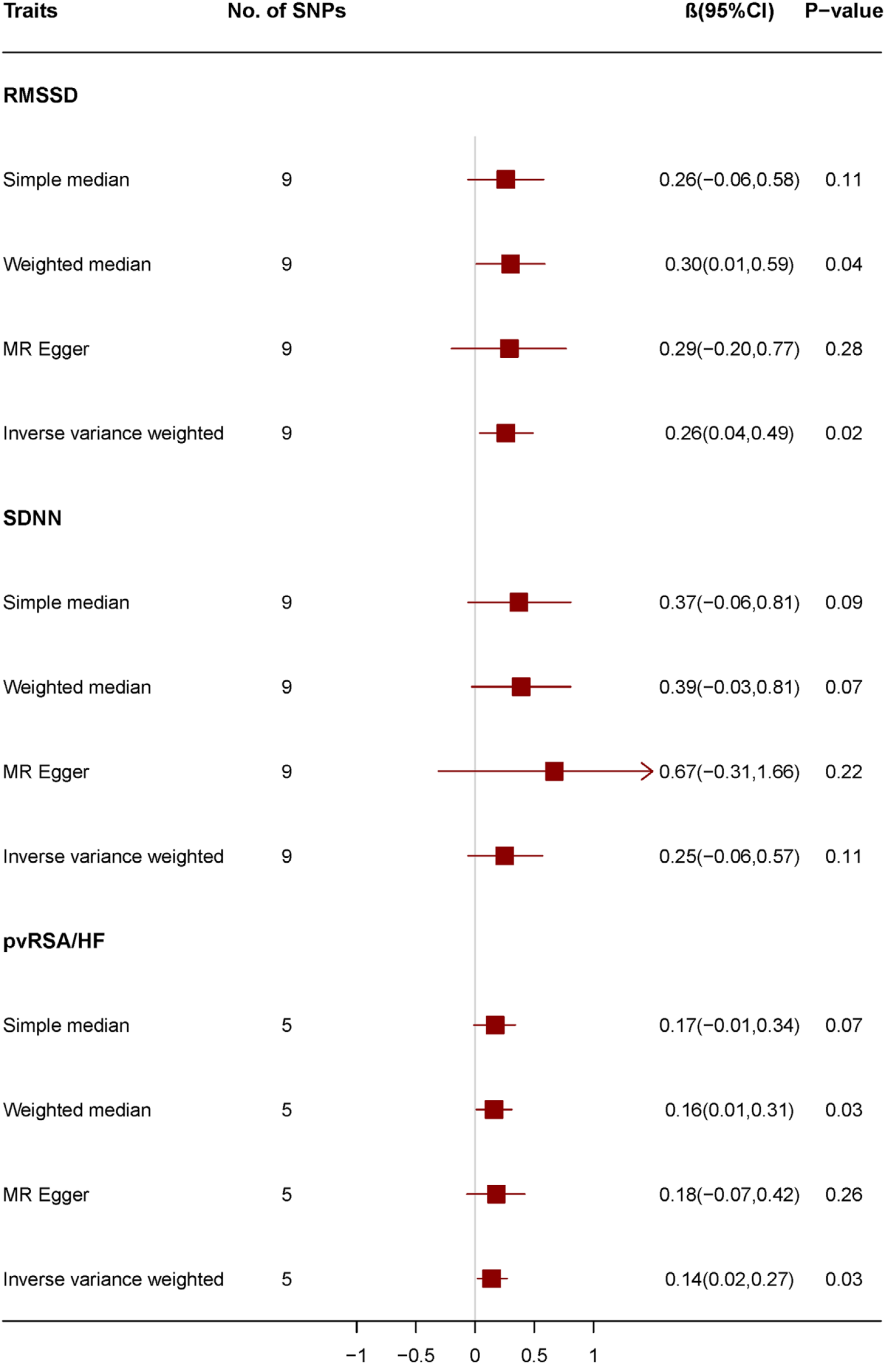
Mendelian randomization for associations between traits of heart rate variability and white matter hyperintensity. Abbreviations: pvRSA/HF, the peak‐valley respiratory sinus arrhythmia or high frequency power; RMSSD, the root mean square of the successive differences of interbeat intervals; SDNN, the standard deviation of the normal‐to‐normal interbeat intervals; SNP, single nucleotide polymorphisms

Genetically predicted RMSSD and pvRSA/HF were suggestively associated with WMH in the IVW analysis (β 0.26, 95% confidence interval [CI] 0.04–0.49, *p* = .02; β 0.14, 95% CI 0.02–0.27, *p* = .03, respectively), which is the principle analysis. The results from the weighted median analysis but not the MR‐Egger regression analysis or the simple median analysis were consistent with the IVW analysis. Genetically predicted SDNN was not significantly associated with WMH in the IVW analysis (β 0.25, 95% CI –0.06 to 0.57, *p* = .11). MR‐Egger regression and MR‐PRESSO analysis provided no evidence of directional pleiotropy for the associations of RMSSD and pvRSA/HF with WMH (intercept = –0.001, *p* = .91; intercept = –0.004, *p* = .79, respectively in MR‐Egger regression, *p*>.05 in MR‐PRESSO analysis) (Figure 2; Supplement Tables S2 and S4). The MR effect size, scatter plot, and leave‐one‐out analysis of relationship between RMSSD, pvRSA/HF and white matter hyperintensity have been shown in supplement data (supplement Figures S1–S6).

### 3.3 The association between HRV and SVS

**FIGURE 3:**
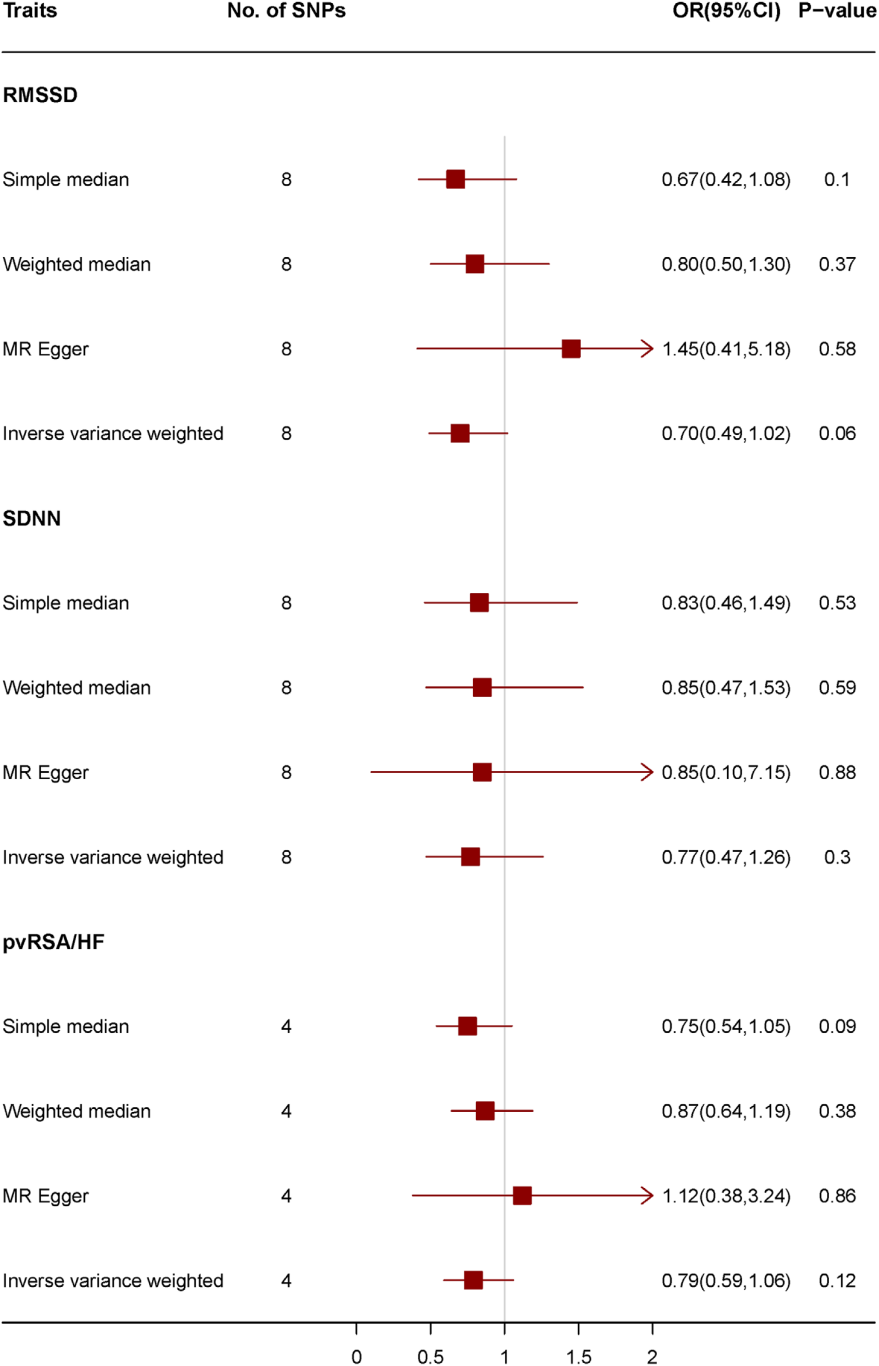
Mendelian randomization for associations between traits of heart rate variability and small vessel stroke. Abbreviations: pvRSA/HF, the peak‐valley respiratory sinus arrhythmia or high frequency power; RMSSD, the root mean square of the successive differences of interbeat intervals; SDNN, the standard deviation of the normal‐to‐normal interbeat intervals; SNP, single nucleotide polymorphisms.

Genetically predicted traits of HRV were not significantly associated with SVS (Figure 3; Supplement Tables S3 and S4).

## 4 DISCUSSION

In this study, we found that HRV (RMSSD, pvRSA/HF) is suggestively associated with an increased risk of WMH but not SVS. Increased HRV was suggested to be correlated with blunted nocturnal heart rate dipping, which may represent a state of sympathetic overdrive and was a response to mental or physical stress, cardiac or noncardiac disease, and other situations.^[^ ^19^ ^]^ HRV is reported to be associated with several neurologic diseases, such as stroke, multiple sclerosis, muscular dystrophies, and Parkinson's disease. In previous studies, increased HRV was also associated with resistant hypertension and cardiovascular events.^[^ ^20^, ^21^, ^22^ ^]^ The pathogenetic mechanisms of cSVD remain largely unknown despite its harmful consequences on stroke, cognitive impairment, and gait disorder. It is suggested that autosomal imbalance plays a role in disease onset; however, previous observational studies^[^ ^5^, ^6^ ^]^ have revealed paradoxical results.

Some previous observational studies reported that increased HRV is associated with cSVD and cSVD progression.^[^ ^5^, ^23^ ^]^ HRV was also increased in obstructive sleep apnea patients with WMH compared with patients without WMH.^[^ ^24^ ^]^ Paradoxically, another study showed that increased HRV was associated with a lower risk of cSVD (quantified by the cSVD score).^[^ ^6^ ^]^ Other studies failed to identify a link between HRV and WMH in patients with community‐dwelling people.^[^ ^25^ ^]^ Our MR study, which decreased confounding bias and reverse causation, showed that HRV is suggestively associated with an increased risk of WMH but not SVS.

The following mechanisms could be responsible for the causal relationship between HRV and WMH: first, higher HRV was associated with an increase in pulsatile flow on the vascular wall, which may impose mechanical stress on the endothelium, inducing endothelium dysfunction over a long time course^[^ ^26^ ^]^; second, the vascular architecture of the deep brain regions lacks anastomoses; therefore, impaired autoregulation in the rigid vessels could contribute to hypoperfusion, leading to brain damage due to impaired vasodilation.^[^ ^2^ ^]^ In this study, we failed to demonstrate a causal relationship between HRV and SVS. To make a control, there is also no relationship between HRV and other stroke subtypes and no relationship between other heart rate trait (resting heart rate) and WMH and SVS (supplement Tables SV and SVI). WMH and SVS are different imaging manifestations of the same general type of disease. Whether differing pathogeneses play a role in the correlation between HRV and cSVD needs to be evaluated in the future.

Our study provides a genetic view of the influence of autosomal imbalance on WMH. A previous study suggested the use of HRV to detect white matter hyperintensities of presumed vascular origin in community‐dwelling older adults.^[^ ^27^ ^]^ However, the present results from observational studies are controversial. This study showed a suggestively significant relationship from a genetic perspective, which supports the practical use of HRV detection. Moreover, HRV and autonomic imbalance are suspected to have an effect on the association between the apnea‐hypopnea index and cSVD,^[^ ^24^ ^]^ and our results could provide genetic evidence for further research.

This study has several strengths. First, this MR study reduced the number of confounding factors and avoided reverse causation. Second, the correlation between HRV and cSVD from observational studies are paradoxical, and this study showed that increased HRV was suggestively correlated with WMH risk from a genetic perspective. Third, the data we used were from large‐scale GWASs, providing a sufficient sample size for high statistical power.

There are several limitations in this study. First, the HRV traits were measured during rest or a 10‐h daytime recording. However, previous observational studies found that HRV during the nighttime, instead of HRV during the daytime, was associated with WMH and cerebrovascular results.^[^ ^22^, ^27^ ^]^ HRV during the daytime is easily affected by blood pressure, emotion and physical activity changes, and these confounding factors could not be avoided by observational studies. MR studies can reduce confounding bias and suggest a significant relationship. Further studies of HRV at night and cSVD are still needed. Second, the genetic variants associated with the traits explain only a small fraction of the variation in the risk factors, and we cannot exclude that the lack of significant associations of HRV with SVS may be due to insufficient statistical power. Third, the result of the MR‐Egger is not significant in this study. The sensitivity analysis does not affect the result in principle analysis, probably because the poor statistic power of MR‐Egger. Fourth, a lack of analysis of gene–environment interaction exists in this study, further gene–environment interaction analysis could better clarify the correlation between HRV and cSVD.

## 5 SUMMARY

This study provides genetic support for a suggestive causal effect of HRV (RMSSD, pvRSA/HF) on WMH, but not SVS.

## FUNDING

Not applicable.

## CONFLICTS OF INTEREST

None of the authors have any conflicts of interest associated with this study.

## AUTHORS’ CONTRIBUTIONS

All authors contributed to the study conception and design, which was mainly conducted by Dongsheng Fan, Tao Huang, and Danyang Tian. Material preparation and data collection were performed by Danyang Tian. Statistical analysis was prepared by Danyang Tian, Linjing Zhang, Dongsheng Fan, and Zhenhuang Zhuang. The first draft of the manuscript was written by Danyang Tian and all authors commented on previous versions of the manuscript. All authors read and approved the final manuscript.

## AVAILABILITY OF DATA AND MATERIAL

The data we used in this article are obtained from public GWAS database.

## Supporting information

SUPPORTING INFORMATION
