# Supplementary material for: A two‐sample Mendelian randomization analysis of heart rate variability and cerebral small vessel disease
Source: J Clin Hypertens (Greenwich). 2021 Jul 1;23(8):1608–14. doi: 10.1111/jch.14316 (PMC8678680; doi:10.1111/jch.14316)
Supplement: Supplementary file 1 — SUPPORTING INFORMATION [file JCH-23-1608-s001.pdf]

Heart rate variability and cerebral small vessel disease: a two-sample Mendelian randomization analysis

Danyang Tian, PhD1, Linjing Zhang, PhD1, Yu Fu, PhD1, Zhenhuang Zhuang2, MD, Tao Huang, PhD2[#], Dongsheng Fan, PhD1[#]

1. Department of Neurology, Pecking University Third Hospital, Beijing, China.

2. Department of Epidemiology & Biostatistics, School of Public Health, Peking University, Beijing, China.

[#]These authors contributed equally to this work and should be considered co-corresponding authors

Supplemental Material

Supplement table Ⅰ: Characteristics of selected SNPs.
Supplement table II : Association of heart rate variability with white matter hyperintensity,
Supplement table Ⅲ: Association of heart rate variability with small vessel stroke.
Supplement tableⅣ: Mendelian randomization of traits on heart rate variability and cerebral small vascular disease.
Supplement tableⅤ: Mendelian randomization of traits on heart rate variability and cardiovascular disease and stroke.
Supplement table VI: Mendelian randomization of traits on resting heart rate and cSVD, cardiovascular disease and stroke.
Supplement figure Ⅰ: MR effect size for RMSSD on white matter hyperintensity.
Supplement figure Ⅱ: Scatter plot for RMSSD on white matter hyperintensity.
Supplement figure Ⅲ: Leave-one-out analysis for RMSSD on white matter hyperintensity.
Supplement figure Ⅳ: MR effect size for pvRSA/HF on white matter hyperintensity.
Supplement figure Ⅴ: Scatter plot for pvRSA/HF on white matter hyperintensity.
Supplement figure Ⅵ: Leave-one-out analysis for pvRSA/HF on white matter hyperintensity.

Supplemental methods

Supplement table Ⅰ: Characteristics of selected SNPs.

Traits	SNP	Position	Nearest gene	Risk allele	MAF	Other allele	effect	SE	p-value	Variance explained	F statistic
pvRSA/HF	rs10842383	12:24663234	LINC00477 (C12orf67)	C	0.87	T	-0.12	0.01	1.2E-25	0.002919	91
pvRSA/HF	rs12974440	19:5845386	NDUA11	A	0.07	G	-0.24	0.02	1.91E-41	0.005555	165
pvRSA/HF	rs1351682	12:33490042	SYT10	G	0.44	A	-0.07	0.01	5.7E-15	0.002143	66
pvRSA/HF	rs236349	6:36928543	PPIL1	G	0.65	A	-0.07	0.01	3.16E-15	0.001744	59
pvRSA/HF	rs4262	7:93389364	GNG11	C	0.39	T	-0.05	0.01	1.84E-11	0.000799	25
RMSSD	rs10842383	12:24663234	LINC00477 (C12orf67)	C	0.86	T	-0.07	0.01	2.45E-29	0.002708	117
RMSSD	rs12974991	19:5845584	NDUA11	A	0.08	G	-0.12	0.01	4.57E-46	0.004843	210
RMSSD	rs180238	7:93388383	GNG11	C	0.33	T	-0.03	0.00	7.99E-16	0.001624	72
RMSSD	rs1812835	15b:71294557	NEO1	A	0.42	C	-0.03	0.00	5.18E-10	0.000879	39
RMSSD	rs2052015	14b:71556806	RGS6	T	0.17	C	-0.04	0.01	3.56E-10	0.000791	36
RMSSD	rs236349	6:36928543	PPIL1	G	0.66	A	-0.04	0.00	9.1E-17	0.001634	77
RMSSD	rs36423	14a:71422955	RGS6	T	0.13	G	-0.04	0.01	5.36E-11	0.000978	44
RMSSD	rs6123471	20:36273570	KIAA1755	T	0.53	C	-0.02	0.00	1.3E-8	0.000769	36
RMSSD	rs7980799	12:33468257	SYT10	A	0.39	C	-0.04	0.00	3.19E-20	0.002146	95
SDNN	rs10842383	12:24663234	LINC00477 (C12orf67)	C	0.86	T	-0.05	0.00	9.33E-31	0.003129	150
SDNN	rs12980262	19:5844058	NDUA11	A	0.08	G	-0.06	0.01	2.3E-23	0.002167	100
SDNN	rs1384598	12:33514166	SYT10	T	0.43	A	-0.02	0.00	7.37E-13	0.001240	59
SDNN	rs236349	6:36928543	PPIL1	G	0.65	A	-0.03	0.00	3.7E-25	0.002350	121
SDNN	rs2529471	14c:71883022	RGS6	C	0.43	A	-0.02	0.00	1.88E-12	0.000987	49
SDNN	rs2680344	15a:71440538	HCN4	A	0.78	G	-0.02	0.00	4.88E-11	0.000700	36
SDNN	rs36423	14a:71422955	RGS6	T	0.13	G	-0.03	0.01	6.25E-13	0.000903	44
SDNN	rs4262	7:93389364	GNG11	C	0.39	T	-0.03	0.00	4.26E-17	0.001775	87
SDNN	rs4899412	14b:71534015	RGS6	T	0.25	C	-0.03	0.00	3.13E-13	0.000875	42

pvRSA/HF: the peak-valley respiratory sinus arrhythmia or high frequency power; RMSSD: the root mean square of the successive differences of inter beat intervals; SDNN: the standard deviation of the normal-to-normal inter beat intervals; SNP: single nucleotide polymorphism; MAF: minor allele frequency.

Supplement table Ⅱ: Association of heart rate variability with white matter hyperintensity,

Traits	SNP	Position	Nearest gene	Risk allele	Other allele	BETA	SE	P-value	MAF
pvRSA/HF	rs10842383	12:24663234	LINC00477(C12orf67)	C	T	-0.01837	0.018122	0.602047	0.14799
pvRSA/HF	rs12974440	19:5845386	NDUA11	G	A	0.040349	0.022772	0.327391	0.087309
pvRSA/HF	rs1351682	12:33490042	SYT10	G	A	-0.01918	0.012866	0.420467	0.53558
pvRSA/HF	rs236349	6:36928543	PPIL1	A	G	-0.01108	0.013317	0.67565	0.65135
pvRSA/HF	rs4262	7:93389364	GNG11	C	T	-0.01215	0.012939	0.631991	0.58202
RMSSD	rs10842383	12:24663234	LINC00477(C12orf67)	C	T	-0.01837	0.018122	0.602047	0.14799
RMSSD	rs12974991	19:5845584	NDUA11	G	A	0.040399	0.02277	0.326672	0.087326
RMSSD	rs180238	7:93388383	GNG11	C	T	-0.01132	0.013387	0.670132	0.65205
RMSSD	rs1812835	15b:71294557	NEO1	C	A	0.028364	0.012852	0.209486	0.41277
RMSSD	rs2052015	14b:71556806	RGS6	C	T	0.008282	0.017684	0.823576	0.16001
RMSSD	rs236349	6:36928543	PPIL1	A	G	-0.01108	0.013317	0.67565	0.65135
RMSSD	rs36423	14a:71422955	RGS6	T	G	0.004271	0.019815	0.921946	0.88031
RMSSD	rs6123471	20:36273570	KIAA1755	T	C	-0.00253	0.012823	0.928749	0.4633
RMSSD	rs7980799	12:33468257	SYT10	A	C	-0.01006	0.01305	0.700522	0.58397
SDNN	rs10842383	12:24663234	LINC00477(C12orf67)	C	T	-0.01837	0.018122	0.602047	0.14799
SDNN	rs12980262	19:5844058	NDUA11	G	A	0.039363	0.02271	0.339426	0.087506
SDNN	rs1384598	12:33514166	SYT10	T	A	-0.01849	0.012866	0.439635	0.5337
SDNN	rs236349	6:36928543	PPIL1	A	G	-0.01108	0.013317	0.67565	0.65135
SDNN	rs2529471	14c:71883022	RGS6	A	C	-0.01368	0.012883	0.582591	0.41749
SDNN	rs2680344	15a:71440538	HCN4	A	G	-0.00185	0.015367	0.957296	0.22353
SDNN	rs36423	14a:71422955	RGS6	T	G	0.004271	0.019815	0.921946	0.88031
SDNN	rs4262	7:93389364	GNG11	C	T	-0.01215	0.012939	0.631991	0.58202
SDNN	rs4899412	14b:71534015	RGS6	T	C	-0.0169	0.014586	0.54446	0.7469

pvRSA/HF: the peak-valley respiratory sinus arrhythmia or high frequency power; RMSSD: the root mean square of the successive differences of inter beat intervals; SDNN: the standard deviation of the normal-to-normal inter beat intervals; SNP: single nucleotide polymorphism; MAF: minor allele frequency.

Supplement table Ⅲ: Association of heart rate variability with small vessel stroke.

Traits	SNP	Position	Nearest gene	Risk allele	Other allele	BETA	SE	P-value	MAF
pvRSA/HF	rs10842383	12:24663234	LINC00477(C12orf67)	t	c	-0.0121	0.0243	0.6191	0.143
pvRSA/HF	rs1351682	12:33490042	SYT10	a	g	-0.0481	0.0196	0.01439	0.628
pvRSA/HF	rs236349	6:36928543	PPIL1	a	g	6.00E-04	0.0172	0.973	0.3628
pvRSA/HF	rs4262	7:93389364	GNG11	t	c	-0.0238	0.0183	0.1931	0.6328
RMSSD	rs10842383	12:24663234	LINC00477(C12orf67)	t	c	-0.0121	0.0243	0.6191	0.143
RMSSD	rs180238	7:93388383	GNG11	t	c	-0.0207	0.0184	0.2593	0.6843
RMSSD	rs1812835	15b:71294557	NEO1	a	c	0.0384	0.0177	0.02966	0.3627
RMSSD	rs2052015	14b:71556806	RGS6	t	c	-0.0168	0.023	0.464	0.1636
RMSSD	rs236349	6:36928543	PPIL1	a	g	6.00E-04	0.0172	0.973	0.3628
RMSSD	rs36423	14a:71422955	RGS6	t	g	0.0042	0.0219	0.8475	0.2595
RMSSD	rs6123471	20:36273570	KIAA1755	t	c	0.0144	0.0165	0.3846	0.5402
RMSSD	rs7980799	12:33468257	SYT10	a	c	0.0333	0.0208	0.1105	0.3454
SDNN	rs10842383	12:24663234	LINC00477(C12orf67)	t	c	-0.0121	0.0243	0.6191	0.143
SDNN	rs1384598	12:33514166	SYT10	a	t	-0.0419	0.02	0.0361	0.6298
SDNN	rs236349	6:36928543	PPIL1	a	g	6.00E-04	0.0172	0.973	0.3628
SDNN	rs2529471	14c:71883022	RGS6	a	c	0.0215	0.017	0.2047	0.594
SDNN	rs2680344	15a:71440538	HCN4	a	g	0.024	0.0206	0.2431	0.7556
SDNN	rs36423	14a:71422955	RGS6	t	g	0.0042	0.0219	0.8475	0.2595
SDNN	rs4262	7:93389364	GNG11	t	c	-0.0238	0.0183	0.1931	0.6328
SDNN	rs4899412	14b:71534015	RGS6	t	c	-0.0084	0.0195	0.6645	0.2482

pvRSA/HF: the peak-valley respiratory sinus arrhythmia or high frequency power; RMSSD: the root mean square of the successive differences of inter beat intervals; SDNN: the standard deviation of the normal-to-normal inter beat intervals; SNP: single nucleotide polymorphism; MAF: minor allele frequency.

Supplement table IV: Mendelian randomization of traits on heart rate variability and cerebral small vascular disease.

	White matter hyperintensity				Small vessel stroke			
Trait/Method	number of SNPs	BETA	SE	P value	number of SNPs	BETA	SE	P value
**pvRSA/HF**								
Simple median	5	0.165365	0.091538	0.070839	4	-0.28679	0.170761	0.093057
Weighted median	5	0.162406	0.076112	0.032861	4	-0.139	0.158785	0.381356
MR Egger	5	0.17501	0.127281	0.262827	4	0.110213	0.543394	0.858034
Inverse variance weighted	5	0.143029	0.06515	0.028135	4	-0.23782	0.151551	0.116584
(intercept)	5	-0.00381	0.012988	0.788297	4	-0.02823	0.041924	0.570132
MR-PRESSO	5	-	-	0.854045	4	-	-	0.222488
**RMSSD**								
Simple median	9	0.258051	0.16222	0.111667	8	-0.39308	0.240794	0.102591
Weighted median	9	0.303368	0.147773	0.040079	8	-0.21954	0.246649	0.373412
MR Egger	9	0.289416	0.247619	0.280745	8	0.374385	0.64802	0.584456
Inverse variance weighted	9	0.262615	0.114248	0.021525	8	-0.3514	0.18857	0.062393
(intercept)	9	-0.00132	0.010844	0.906332	8	-0.02764	0.023614	0.286120
MR-PRESSO	9	-	-	0.652334	8	-	-	0.587464
**SDNN**								
Simple median	9	0.374816	0.223453	0.093467	8	-0.18711	0.297777	0.529781
Weighted median	9	0.393072	0.214477	0.066847	8	-0.16445	0.301341	0.585262
MR Egger	9	0.672697	0.50316	0.223053	8	-0.16426	1.087676	0.884907
Inverse variance weighted	9	0.254646	0.159399	0.110146	8	-0.2642	0.253153	0.29666
(intercept)	9	-0.0138	0.015755	0.410097	8	-0.00304	0.032076	0.927469
MR-PRESSO	9	-	-	0.221598	8	-	-	0.551339

pvRSA/HF: the peak-valley respiratory sinus arrhythmia or high frequency power; RMSSD: the root mean square of the successive differences of inter beat intervals; SDNN: the standard deviation of the normal-to-normal inter beat intervals; SNP: single nucleotide polymorphism.

Supplement table V : Mendelian randomization of traits on heart rate variability and cardiovascular disease and stroke.

Outcome	Exposure	Method	Number of SNPs	BETA	SE	P-Value
WMH	pvRSA/HF	Inverse variance weighted	5	0.143029	0.06515	0.028135
SVS	pvRSA/HF	Inverse variance weighted	4	-0.23782	0.151551	0.116584
LAS	pvRSA/HF	Inverse variance weighted	5	0.257842	0.121702	0.034122
AIS	pvRSA/HF	Inverse variance weighted	5	0.052737	0.049321	0.284958
ICH	pvRSA/HF	Inverse variance weighted	3	4.053741	1.953996	0.038024
CAD	pvRSA/HF	Inverse variance weighted	5	0.05018	0.071509	0.482848
WMH	RMSSD	Inverse variance weighted	9	0.262615	0.114248	0.021525
SVS	RMSSD	Inverse variance weighted	8	-0.3514	0.18857	0.062393
LAS	RMSSD	Inverse variance weighted	9	0.100713	0.271909	0.711091
AIS	RMSSD	Inverse variance weighted	9	0.091534	0.099242	0.356358
ICH	RMSSD	Inverse variance weighted	8	-0.69905	4.341195	0.872073
CAD	RMSSD	Inverse variance weighted	9	0.081793	0.087307	0.348843
WMH	SDNN	Inverse variance weighted	9	0.254646	0.159399	0.110146
SVS	SDNN	Inverse variance weighted	8	-0.2642	0.253153	0.29666
LAS	SDNN	Inverse variance weighted	8	0.09165	0.3959	0.816928
AIS	SDNN	Inverse variance weighted	9	0.107385	0.117392	0.360319
ICH	SDNN	Inverse variance weighted	6	7.777066	5.210529	0.13555
CAD	SDNN	Inverse variance weighted	9	0.098631	0.136389	0.469585

WMH: white matter hyperintensity; SVS: small vessel stroke; LAS: large artery atherosclerotic stroke; AIS: acute ischemic stroke; ICH: intracerebral hemorrhage; CAD: cardiovascular disease. pvRSA/HF: the peak-valley respiratory sinus arrhythmia or high frequency power; RMSSD: the root mean square of the successive differences of inter beat intervals; SDNN: the standard deviation of the normal-to-normal inter beat intervals; SNP: single nucleotide polymorphism.

Supplement table VI: Mendelian randomization of traits on resting heart rate and cSVD, cardiovascular disease and stroke.

Outcome	Exposure	Method	Number of SNPs	BETA	SE	P-Value
WMH	Resting Heart Rate	Inverse variance weighted	61	-0.00567	0.006809	0.404797
SVS	Resting Heart Rate	Inverse variance weighted	57	-0.00946	0.007435	0.203245
LAS	Resting Heart Rate	Inverse variance weighted	61	0.021041	0.009436	0.025763
AIS	Resting Heart Rate	Inverse variance weighted	62	0.012572	0.004492	0.005127
ICH	Resting Heart Rate	Inverse variance weighted	41	0.025238	0.238658	0.915781
CAD	Resting Heart Rate	Inverse variance weighted	62	0.005833	0.00641	0.362818
CES	Resting Heart Rate	Inverse variance weighted	61	0.019768	0.010351	0.056166

WMH: white matter hyperintensity; SVS: small vessel stroke; LAS: large artery atherosclerotic stroke; AIS: acute ischemic stroke; ICH: intracerebral hemorrhage; CAD: cardiovascular disease. SNP: single nucleotide polymorphism.

Supplement figure Ⅰ: MR effect size for RMSSD on white matter hyperintensity.

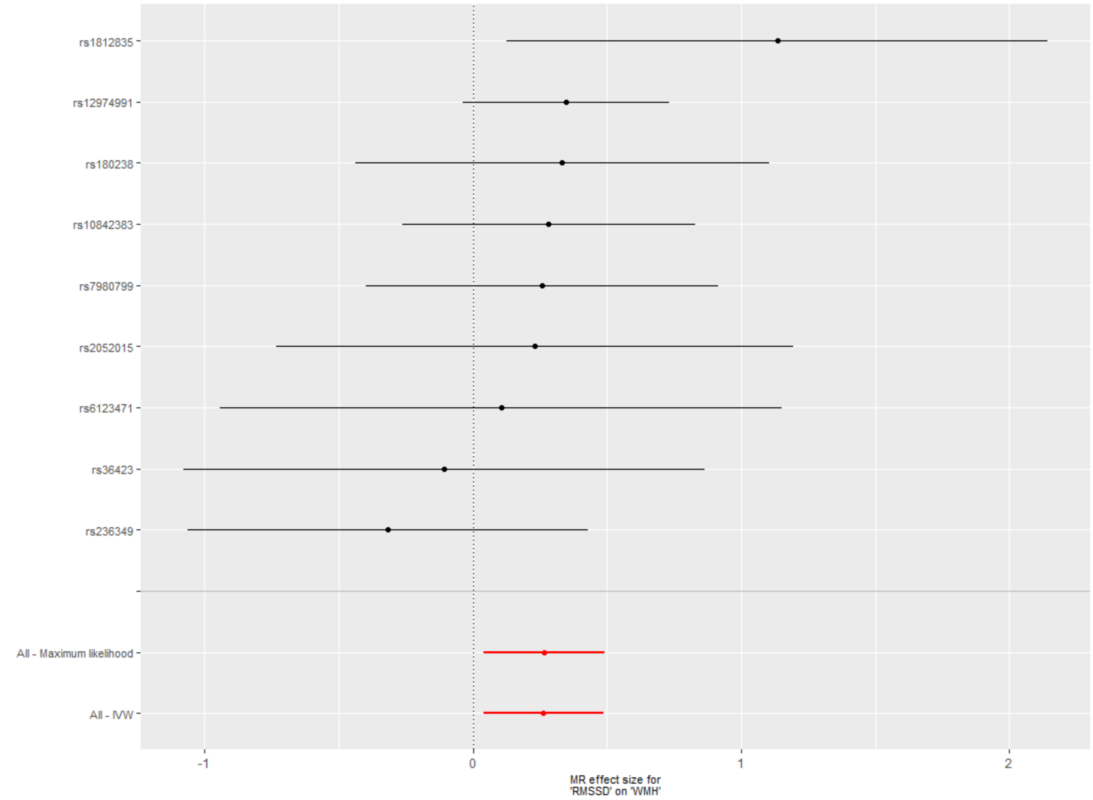

Supplement figure Ⅱ: Scatter plot for RMSSD on white matter hyperintensity.

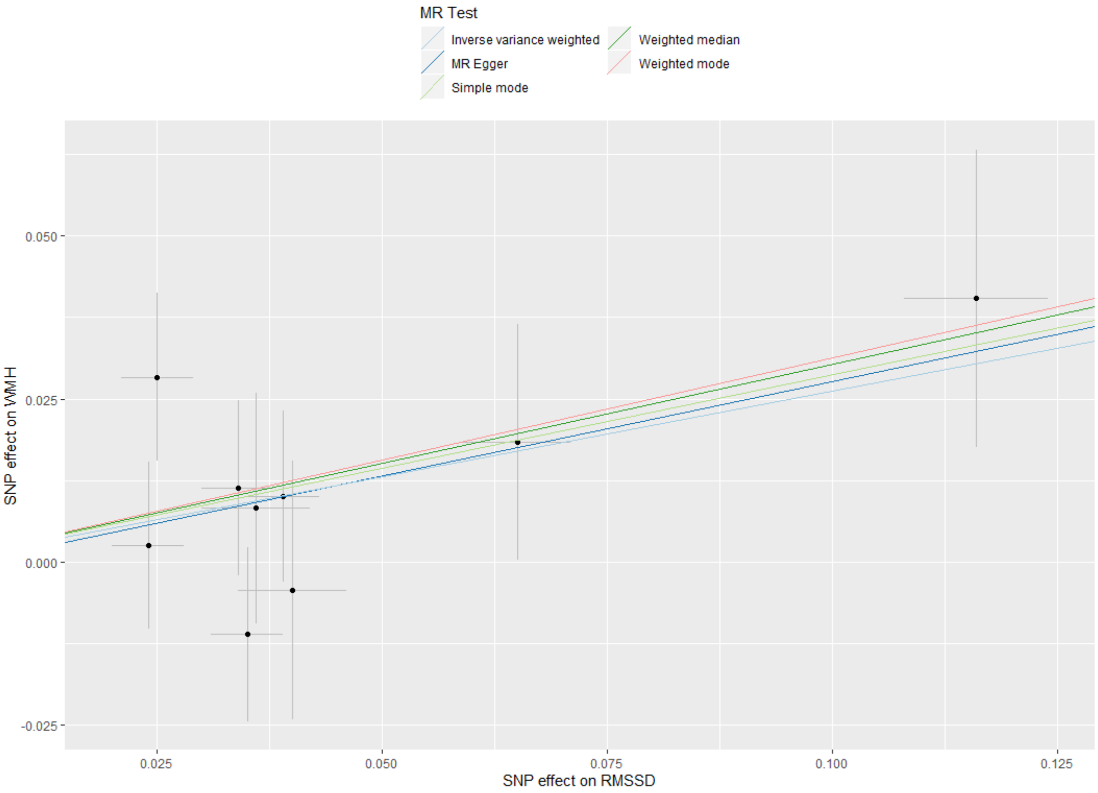

Supplement figure Ⅲ: Leave-one-out analysis for RMSSD on white matter hyperintensity.

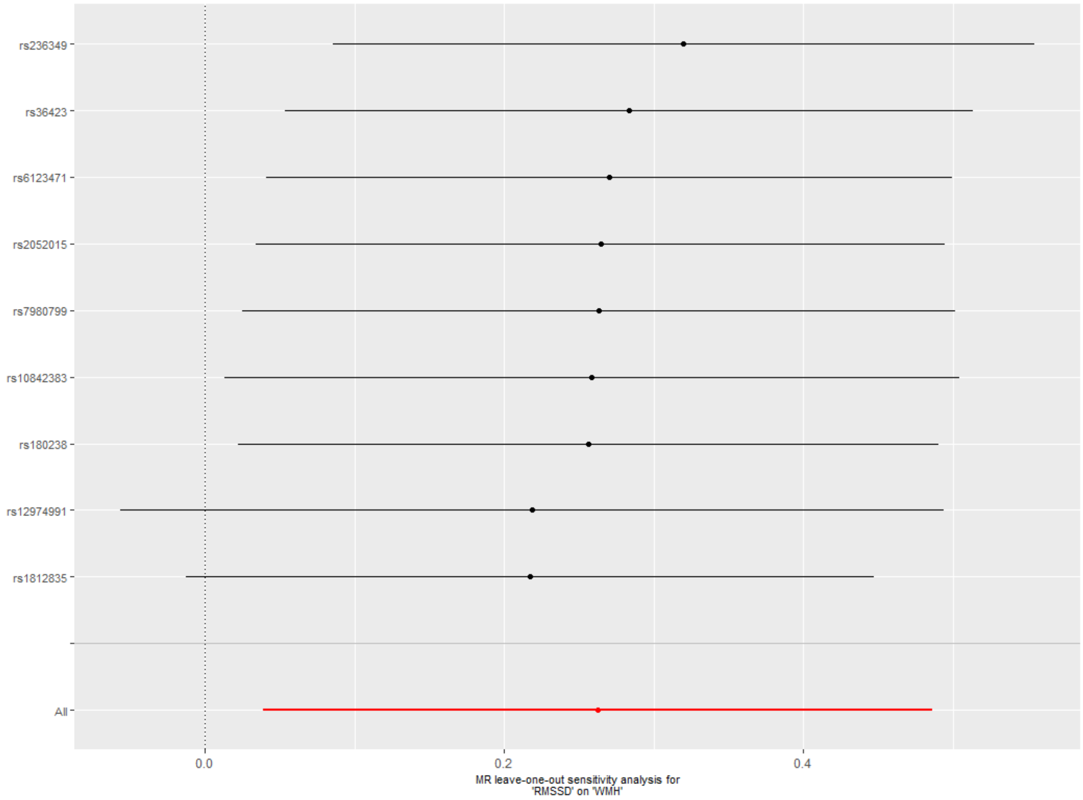

Supplement figure Ⅳ: MR effect size for pvRSA/HF on white matter hyperintensity.

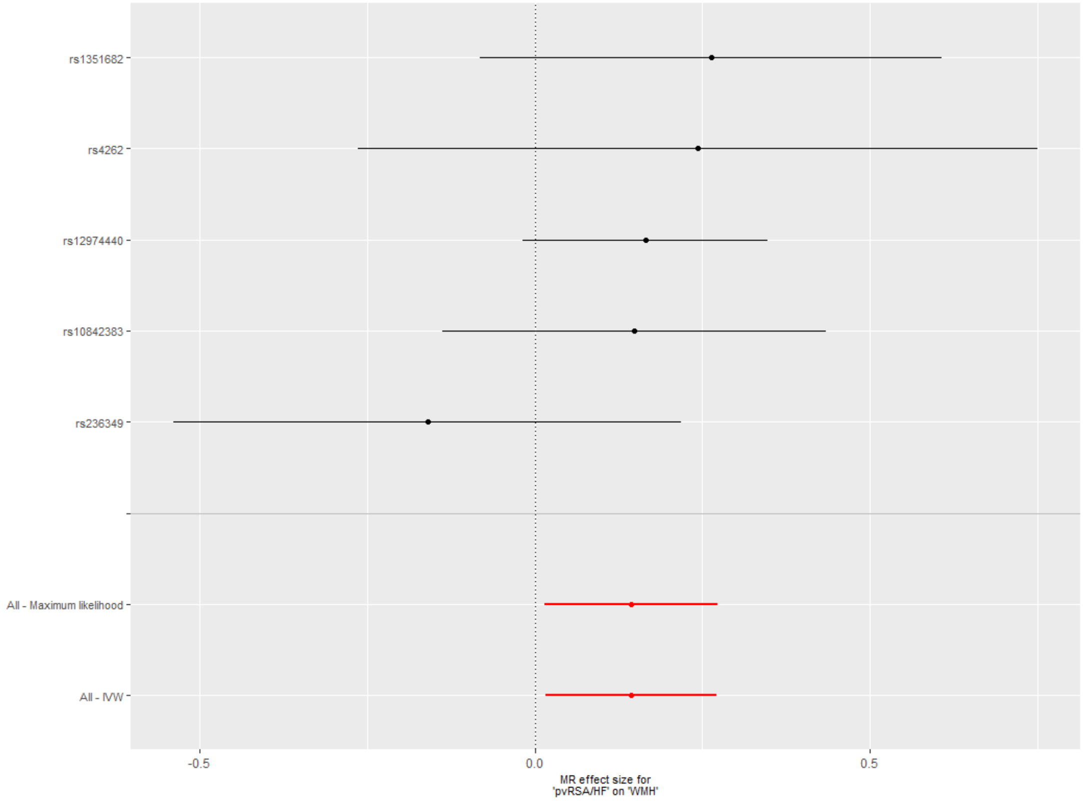

Supplement figure V : Scatter plot for pvRSA/HF on white matter hyperintensity.

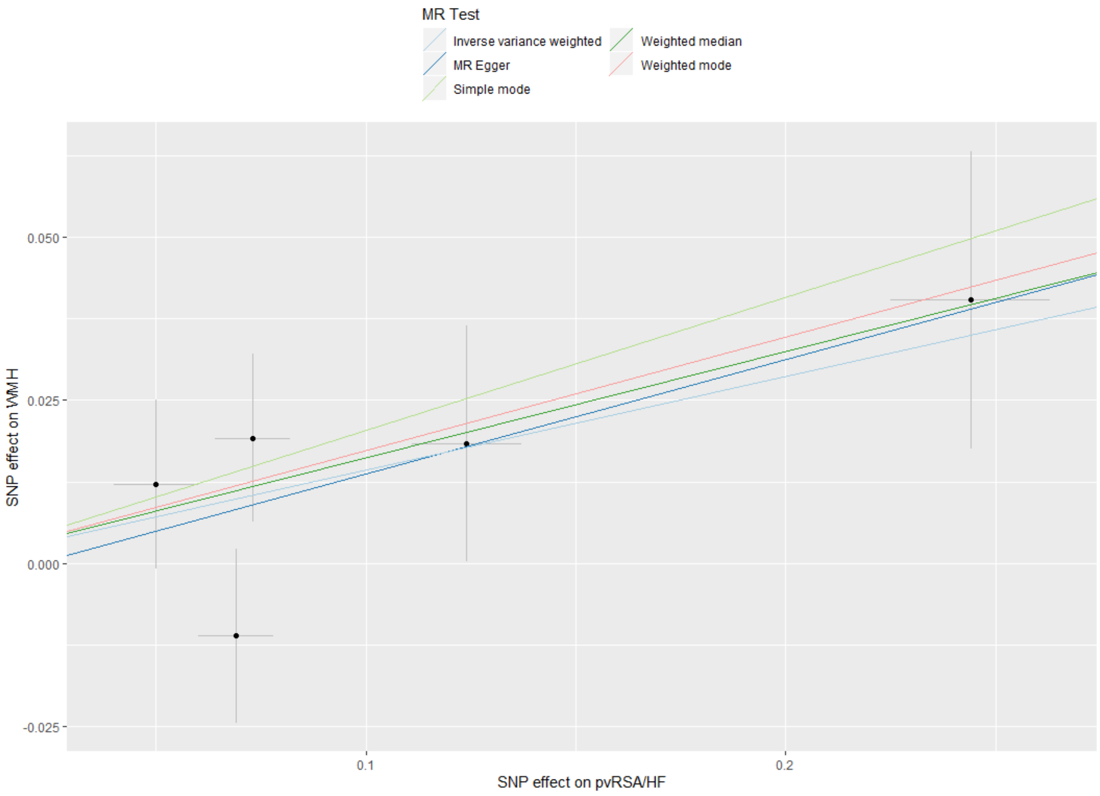

Supplement figure VI: Leave out analysis for pvRSA/HF on white matter hyperintensity.

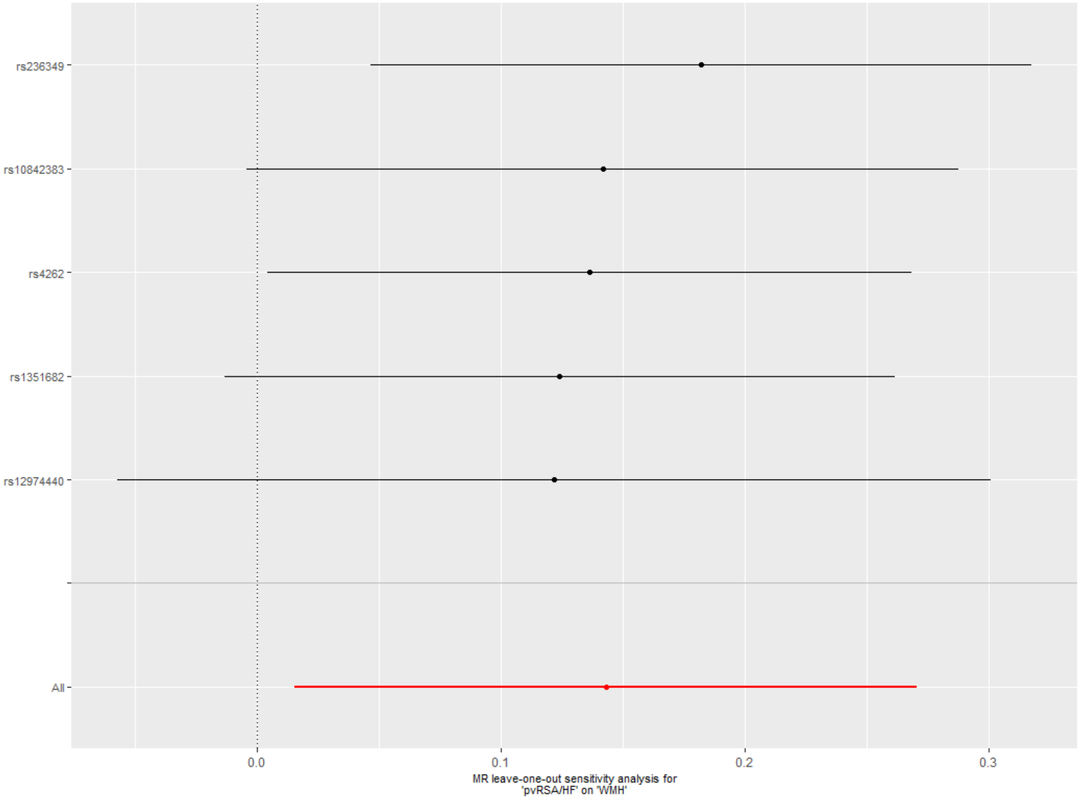

Supplemental methods

Study design and genetic analysis of MEGASTROKE study

In the MEGASTROKE study, about 8 million SNPs and indels with minor-allele frequency (MAF) ⩾0.01 were tested in up to 67,162 stroke cases and 454,450 controls for association with stroke. One analysis involved European participants only (40,585 cases; 406,111 controls), and a second involved participants of European, East Asian (17,369; 28,195), African (5,541; 15,154), South Asian (2,437; 6,707), mixed Asian (365; 333), and Latin American (865; 692) ancestry (Fig. 1). Participants were drawn from 29 studies with genome-wide genotypes imputed to 1000 Genomes Project (1000G) phase 1v3 or similar14 (MEGASTROKE consortium; Supplementary Note and Supplementary Tables 1 and 2). Ancestry-specific meta-analyses and subsequent fixed-effects transancestral meta-analyses and MANTRA transancestral meta-analyses were conducted15. Analyses were performed for any stroke (AS), comprising ischemic stroke, ICH, and stroke of unknown or undetermined type (n = 67,162); any ischemic stroke (AIS) regardless of subtype (n = 60,341); and ischemic stroke subtypes (LAS, n = 6,688; CES, n = 9,006; SVS, n = 11,710).

All studies used imputed genotypes based on at least the 1000G phase 1 multiancestral reference panel and conducted logistic regression analyses (or Cox regression for longitudinal population-based cohort studies) for five stroke traits (AS, AIS, LAS, CES, and SVS) with all measured and imputed genetic variants in dosage format by using appropriate software under an additive genetic model with a minimum of sex and age as covariates. Before ancestry-specific meta-analysis, QC was performed on each study by two independent researchers following a standardized protocol. Marker names and alleles were harmonized across studies. Meta-analyses were restricted to autosomal biallelic markers from the 1000G phase 1 v3. Duplicate markers were removed from each study. P–Z plots, QQ plots and allele-frequency-plots were constructed for each study. After visual inspection, analysis and QC were repeated if deemed necessary. QC was conducted independently for all participating studies in at least two sites. Individual study-level filters were set to remove extreme effect values (β >5 or β <–5), rare SNPs (MAF <0.01) and variants with low imputation accuracy (oevar_imp or info score <0.5). The effective allele count was defined as twice the product of the MAF, imputation accuracy (r2, info score or oevar_imp), and number of cases. Variants with an effective allele count <10 were excluded. Genome-wide-association meta-analyses, Conditional analysis, Gene-based analysis were further executed. [1]

Study design and genetic analysis of White matter hyperintensity data on UK Biobank.

The data of white matter hyperintensity (WMH) was available from the UK Biobank. UK Biobank enrolled about 500,000 community-dwelling participants from 2006 to 2010 (aged 40-69 years). Data of questionnaires, interviews, health records, physical measures, biological samples and imaging are collected during this prospective. For WMH, the imaging information were collected form the MRI data. BIANCA tool was used to combine data of T1, T2 and FLAIR, calculating the total volume of WMH from the voxels exceeding a probability of 0.9 of being WMH and located within a white matter mask. Obtained values were adjusted for the total intracranial volume and log transformed because of their skewed distribution.[2]

Genetic analysis: in brief, two closely related arrays from Affymetrix, the UK BiLEVE Axiom array (9.9% of individuals) and the UK Biobank Axiom array, were used to genotype

approximately 805,426 markers with good genome-wide coverage. Phasing was performed using SHAPEIT3 and imputation to a merged HRC reference panel (39,131,578 autosomal SNPs) and UK10K & 1000 Genomes Phase 3 panel was carried out using the IMPUTE4 package. Imputed genotypes were available for 487,442 individuals in this study. From the resulting dataset, Individuals that did not segregate with European samples based on PCA analysis, individuals with high levels heterozygosity and missingness (>5%) and individuals whose reported sex was inconsistent with sex inferred from the genetic data were excluded. In addition, only SNPs imputed from the HRC panel were included in this analysis.[3]

Reference

1 Malik, R. *et al.* Multiancestry genome-wide association study of 520,000 subjects identifies 32 loci associated with stroke and stroke subtypes. *Nature genetics* **50**, 524-537, doi:10.1038/s41588-018-0058-3 (2018).

2 Alfaro-Almagro, F. *et al.* Image processing and Quality Control for the first 10,000 brain imaging datasets from UK Biobank. *NeuroImage* **166**, 400-424, doi:10.1016/j.neuroimage.2017.10.034 (2018).

3 Liu, J., Rutten-Jacobs, L., Liu, M., Markus, H. S. & Traylor, M. Causal Impact of Type 2 Diabetes Mellitus on Cerebral Small Vessel Disease: A Mendelian Randomization Analysis. *Stroke* **49**, 1325-1331, doi:10.1161/strokeaha.117.020536 (2018).
